# Compression neuropathy caused by cancer metastasis to the optic nerve canal

**DOI:** 10.1186/1756-0500-6-546

**Published:** 2013-12-20

**Authors:** Hiroshi Tamai, Kazuyuki Ishida, Kensuke Murakami, Norio Narita, Teiji Tominaga, Nobuo Fuse

**Affiliations:** 1 Department of Ophthalmology, Kesennuma City Hospital, 184 Tanaka, Kesennuma, Miyagi 988-0052, Japan; 2 Department of Ophthalmology, Tohoku University Graduate School of Medicine, 1-1 Seiryo-machi, Aoba-ku, Sendai, Miyagi 980-8574, Japan; 3 Department of Neurosurgery, Kesennuma City Hospital, 184 Tanaka, Kesennuma, Miyagi 988-0052, Japan; 4 Department of Pathology, Tohoku University Hospital, 1-1 Seiryo-machi, Aoba-ku, Sendai, Miyagi 980-8574, Japan; 5 Department of Neurosurgery, Tohoku University Graduate School of Medicine, 1-1 Seiryo-machi, Aoba-ku, Sendai, Miyagi 980-8574, Japan; 6 Department of Integrative Genomics, Tohoku Medical Megabank Organization, Tohoku University, 2-1 Seiryo-machi, Aoba-ku, Sendai, Miyagi 980-8573, Japan

**Keywords:** Cancer, Metastasis, Optic canal

## Abstract

**Background:**

Cancerous cells are known to metastasize to different ocular structures. This happens especially to the choroid in males with lung cancer and females with breast cancer. However, we observed two cases of cancerous metastasis to the optic canal region. Both cases showed only a progressive decrease in vision without any other remarkable ophthalmological symptoms or abnormalities in the affected eye.

**Case presentation:**

Two females, a 60-year-old and a 73-year-old, came to our hospital because of progressive loss of vision. These patients showed no remarkable symptoms or signs in their eyes except visual acuity loss. Several ophthalmoscopic examinations, such as slit lamp microscopy and fundoscopy, showed no abnormal changes in their affected eye but magnetic resonance imaging indicated a massive legion around the optic nerve.

**Conclusion:**

It is possible for cancer to metastasize to the optic canal region and the existence of primary tumors should be considered.

## Background

Primary and secondary orbital tumors, including intraorbital and optic nerve tumors, are uncommon observations in daily medical practice. The primary orbital tumors are solitary fibrous tumors, rhabdomyosarcomas, meningiomas, gliomas and others. Secondary orbital tumors include post-transplantation lymphoproliferative disorders and metastatic rhabdomyosarcomas [1]. Primary optic nerve tumors include meningiomas, gliomas and malignant melanomas, and secondary optic nerve tumors include metastases from breast cancer, leukemia, retinoblastoma and gliomatosis cerebri. An earlier study reported that optic nerve tumors make up 8% of all orbital tumors [2] and metastases to the optic nerve area are rare [2-4].

## Case presentations

**Figure 1:**
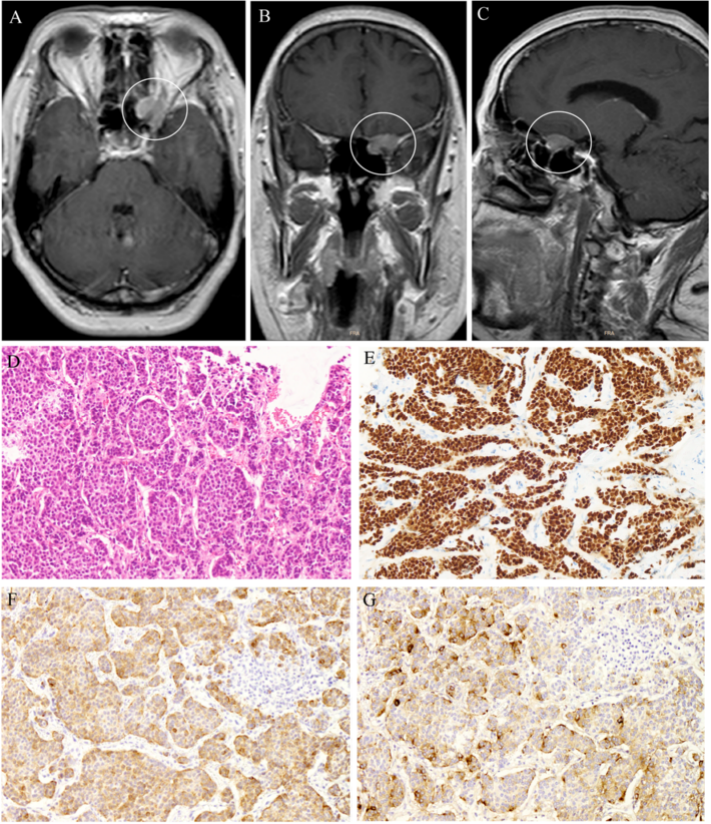
**Magnetic resonance image and pathological examination of a patient with breast cancer that metastasized to the left optic nerve region. A–C**: Tumor mass (white circle) is seen conflicted in the optic canal, surrounding the optic nerve. **D–G**: Histopathological examination of biopsied tissue stained with hematoxylin-eosin (HE) and immunohistochemical staining (original magnification × 200). **D**: Infiltrating ductal carcinoma arranged in cords, cluster and trabeculae (HE staining). **E**: Cytokeratin staining as an epithelial cell marker. **F**: Estrogen receptor staining. **G**: GCDFP-15 staining as an apocrine cell marker.

Case 1: A 60-year-old woman came to our hospital after experiencing a progressive decrease in vision in her left eye of several months duration. Her medical history was not significant except for having diabetes mellitus. The cause of the progressive decrease in vision could not be determined. At her first visit to our hospital, the visual acuity in her right eye was 6/6 but the vision in her left eye had decreased to <6/60. We performed a visual field test and confirmed the visual field loss in her left eye. All ophthalmological tests were normal; e.g., a slit lamp examination did not indicate cornea, pupil or lens abnormalities and a fundoscopic examination, and fluorescent fundus angiography showed no remarkable changes such as optic atrophy or retinal ischemic changes. However, magnetic resonance imaging (MRI) showed a massive lesion in the optic nerve area (Figure 1A–C). Therefore, we suspected a primary orbital tumor and performed a neurosurgical examination. During the neurosurgery to remove the tumor, the mass was seen to surround the optic nerve. The tumor was removed with only aspiration and samples were submitted for histopathological examination. Sections of the aspirated tissue showed that it was a ductal carcinoma (Figure 1D) and immunohistochemical staining of this carcinoma was positive for cytokeratin, an epithelial cell marker for the estrogen receptor, and for GCDFP-15, an apocrine cell marker (Figure 1E–G). These morphological features were compatible with metastasis from a breast carcinoma. We performed positron emission tomography–computed tomography (PET-CT) to screen for cancer lesions throughout the body and confirmed the existence of breast cancer in the left mamma with multiple bone metastases. Seven months after the surgery, her visual field had recovered, although central scotoma had remaind. Thus her visual acuity had not recovered from 6/60.

**Figure 2:**
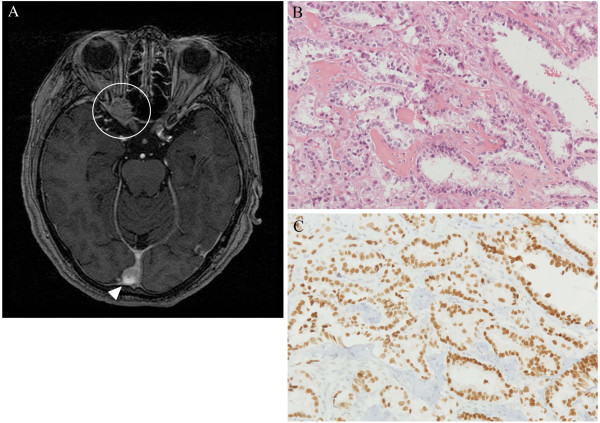
**Magnetic resonance image and histopathological examination of Patient 2 showing breast cancer metastasis to the left optic nerve region. A**: MRI shows one tumor mass in the optic canal surrounding the optic nerve (white circle) and another mass between the occipital lobes (white triangle). **B–C**: Pathological examination of metastasized cancer tissue stained with HE and immunohistochemical staining (original magnification × 200). **B**: Infiltrating adenocarcinoma arranged in irregular-shaped glands (HE staining). **C**: Thyroid transcription factor 1 (TTF-1), the marker of lung adenocarcinoma.

Case 2: A 73-year-old female noted a decrease in the visual acuity of her right eye for a month. She had no remarkable medical history that could cause such a progressive decrease in vision. Her visual acuity in the right eye was counting fingers, and there was a central scotoma in her right visual field with no abnormal changes observed with a slit lamp examination and fundoscopic examination. Her left eye was completely normal. We considered the existence of an orbital tumor and examined her with MRI. MRI showed one mass restricted to the optic nerve region and another mass located between the occipital lobes (Figure 2A). Her left visual field was normal and there was no homonymous hemianopsia. We therefore thought the main cause of her visual loss was the mass at the optic nerve region, and we next performed a surgical biopsy. Histopathological examination of the biopsied tissue showed that it was a metastatic adenocarcinoma (Figure 2B), and immunohistochemical staining showed that it was positive for thyroid transcription factor 1 (TTF-1), which is a marker for lung adenocarcinoma (Figure 2C). During the surgery, the tumor was found to surround the optic nerve, and the upper wall of the superior orbital fissure was replaced by tumorous growth. We also performed PET-CT body scans for cancer screening and confirmed that the primary lesion was in her right lung with multiple bone metastases. Six months after the surgery, her visual acuity recovered to 6/60.

## Conclusion

Both of our cases were secondary tumors as a result of metastases from either a primary breast cancer or lung cancer. We suggest that the growth of the tumor in the optic canal exerted pressure on the optic nerve, which led to the progressive decrease in vision. This was supported by the stationary state and recovery of visual field or visual acuity after surgical removal of the metastatic tumor. Thus, we concluded that the tumor in both patients was secondary and the immunohistochemical staining suggested that the primary cancer was in the breast in Patient 1 and in the lung in Patient 2. PET-CT body scans confirmed the immunohistochemical findings.

We presented our findings on two patients whose only sign and symptom was a progressive decrease in vision. Histopathological examinations of the excised tissues suggested that the retrobulbar tumors were metastases from breast cancer in one patient and lung cancer in the other patient. PET-CT body scans confirmed not only the location of the primary cancer but also the location of the metastatic area. Thus, our examinations of these two patients led not only to stopping the loss of vision but also determining the location of the life-threatening cancer.

## Consent

Written informed consent was obtained from the patients for publication of this Case Report and any accompanying images. A copy of the written consent is available for review by the Executive Editor, Editor-in-Chief of this journal.

## Abbreviations

MRI: Magnetic resonance imaging; PET-CT: Positron emission tomography–computed tomography; TTF-1: Thyroid transcription factor 1.

## Competing interests

The authors have declared that no competing interests exist.

## Authors’ contributions

HT carried out the patient interaction and diagnosis, drafting of the manuscript, final approval of the manuscript. KI carried out the pathological examination. KM carried out the patient interaction and diagnosis, neurosurgical treatment, final approval of the manuscript. NN carried out the patient interaction and diagnosis, neurosurgical treatment, final approval of the manuscript. TT carried out the patient interaction and diagnosis, neurosurgical treatment, final approval of the manuscript. NF carried out the patient interaction and diagnosis, final approval of the manuscript. All authors read and approved the final manuscript.
